# First report of the blood-feeding pattern in *Aedes koreicus*, a new invasive species in Europe

**DOI:** 10.1038/s41598-022-19734-z

**Published:** 2022-09-21

**Authors:** Fabrizio Montarsi, Fausta Rosso, Daniele Arnoldi, Silvia Ravagnan, Giovanni Marini, Luca Delucchi, Roberto Rosà, Annapaola Rizzoli

**Affiliations:** 1 Istituto Zooprofilattico Sperimentale Delle Venezie, Legnaro, Padua, Italy; 2 Research and Innovation Centre, Fondazione Edmund Mach, San Michele All’Adige, TN Italy; 3 Center Agriculture Food Environment, University of Trento, San Michele All’Adige, TN Italy

**Keywords:** Entomology, Ecological epidemiology, Invasive species

## Abstract

*Aedes koreicus* is an invasive mosquito species which has been introduced into several European countries. Compared to other invasive *Aedes* mosquitoes, little is known of its biology and ecology. To determine *Ae. koreicus*’ vectorial capacity, it is essential to establish its feeding patterns and level of anthropophagy. We report on the blood-feeding patterns of *Ae. koreicus*, examining the blood meal origin of engorged females and evaluating the influence of different biotic and abiotic factors on feeding behavior. Mosquitoes were collected in 23 sites in northern Italy by manual aspiration and BG-sentinel traps; host availability was estimated by survey. The source of blood meals was identified using a nested PCR and by targeting and sequencing the cytochrome c oxidase subunit I gene. In total, 352 *Ae. koreicus* engorged females were collected between 2013 and 2020 and host blood meals were determined from 299 blood-fed mosquitoes (84.9%). Eleven host species were identified, with the highest prevalences being observed among roe deer (*Capreolus capreolus*) (*N* = 189, 63.2%) and humans (*N* = 46, 15.4%). Blood meals were mostly taken from roe deer in forested sites and from humans in urban areas, suggesting that this species can feed on different hosts according to local abundance. Two blood meals were identified from avian hosts and one from lizard. *Ae. koreicus’* mammalophilic feeding pattern suggests that it may be a potential vector of pathogens establishing transmission cycles among mammals, whereas its role as a bridge vector between mammals and birds could be negligible.

## Introduction

*Aedes* (Finlaya) *koreicus* (Edwards, 1917) is an invasive species native to Korea, China, Japan, and Russia^1,2^, reported for the first time outside its native range, in Europe (Belgium), in 2008^3^. It is a sibling species of *Aedes japonicus japonicus* (Theobald, 1901), the Asian rock pool or Asian bush mosquito, with which it has been confused in the past^4^. Recent studies predicted the potential for further spread of *Ae. koreicus* throughout temperate regions in Europe given its capacity to tolerate cold temperatures at each life stage^5,6^. The European Centre for Disease Prevention and Control (ECDC)^7^ currently considers the species to be established in Italy^8^, Belgium^9^, Germany^10^, Hungary^11^, Switzerland^12^, and the south of European Russia^2^, and is reported to have been introduced into Slovenia^13^, Austria^14^, and Kazakhstan^15^.

In central Europe, *Ae. koreicus* occurs in a few restricted populations, except for northern Italy, where the species has been spreading quickly and more ubiquitously^16–18^.

Knowledge on the biology and ecology of *Ae. koreicus* is poor and reports are limited to its native range. In addition, this mosquito was previously considered a subspecies of *Ae. j. japonicus*^19^, leading researchers to infer much of the species’ biology and ecology from the better-researched *Ae. j. japonicus*.

*Aedes koreicus* is a container-breeding mosquito that lays eggs in all types of artificial containers and natural holes found in plants and rocks in urban, periurban, and natural environments^20,21^. The eggs have a long survival time, are resistant to desiccation, and can be spread by passive transport such as aircraft, ships, or vehicles^22^. Like other *Aedes* species, *Ae. koreicus* overwinters in cold-resistant eggs that hatch early the following spring^19,23^, with adults occurring for a longer period and partially avoiding larval competition with similar species (i.e., *Aedes albopictus*)^24^. Furthermore, its higher tolerance to the cold^6^ may allow *Ae. koreicus* to establish itself at higher altitudes—where mean temperatures are lower—compared to other *Aedes* species, such as *Ae. albopictus*.

Limited, outdated literature is available on the vector competence of *Ae. koreicus*. Experimental transmission of Japanese encephalitis virus has been demonstrated^25,26^, and it has also been isolated from wild-caught mosquitoes^27^. *Aedes koreicus* has been experimentally proven to be vector-competent for the dog heartworm, *Dirofilaria immitis*^28,29^. Moreover, recent studies under laboratory conditions have reported its potential vector competence for chikungunya and Zika viruses, but its transmission efficiency is influenced by temperature^30,31^.

To assess the potential vectorial capacity of a mosquito species for zoonotic pathogens, it is crucial to evaluate its feeding behavior. Variations in mosquito feeding patterns can be influenced by several factors, as species-specific host preference, environmental conditions, and host availability. This latter is particularly important, but rarely assessed due to the difficulties inherent in obtaining exhaustive quantitative estimates of each individual host species^32^.

This study aims to provide a first evaluation of the feeding patterns of *Ae. koreicus* in a recently invaded mountain area in Europe*.* In previous literature *Ae. koreicus* was reported to present mainly daytime blood-feeding behavior on either domestic animals or humans, but the evidence was anecdotal and no blood meal analyses were performed^1,33^. Likewise, residents in northern Italy observed diurnal activity among *Ae. koreicus,* as subsequently confirmed by Montarsi et al.^16^. However, it remains unclear whether they have a preference for humans or present opportunistic feeding behavior. We conducted field collections of engorged mosquitoes in northern Italy and used molecular techniques to identify the blood meal host species.

## Results

### Collection of Aedes koreicus and blood meal analysis

In total, 352 engorged *Ae. koreicus* females were collected during 112 sampling sessions between 2013 and 2020. Most were collected in 2020 in Trento Province (Table 1 and Supplementary Table S1 online). Of these, 299 blood meals were identified to host species (84.9%). The collection method for most of the engorged mosquitoes was by aspiration (*N* = 338; 96%) (Supplementary Table S1 online).

**Table 1:** Number of identified blood meals and engorged females (between brackets) by year and Province.

Province	2013	2014	2015	2016	2017	2018	2019	2020	Total
Belluno	23 (37)	5 (6)	3 (3)						31 (46)
Trento		1 (1)	8 (21)	36 (36)	4 (14)	78 (80)	1 (2)	140 (152)	268 (306)
Total	23 (37)	6 (7)	11 (24)	36 (36)	4 (14)	78 (80)	1 (2)	140 (152)	299 (352)

The majority of analyzed mosquitoes fed on wild ungulates (chamois, roe deer, and red deer; in total *N* = 238, 79.6%) or humans (*N* = 46, 15.4%) (Table 2). Average densities (animals/hectare) across all sites, based on wildlife census data provided by the provincial forestry offices (Table 2), were estimated to be 0.09, 0.09, and 0.21 for chamois, roe deer, and red deer, respectively. Overall, eleven unique species were identified. In each sample, only one host species was identified and no mixed blood meals were found.

**Table 2:** Identified blood meals per host species in each sampling site (2013–2020).

			N. of blood meal sources (% of total blood meals)										
ID site	Blood meal identified	Main available hosts (number of individuals/hectare)*	Human (*Homo sapiens*)	Dog (*Canis lupus familiaris*)	Fox (*Vulpes vulpes*)	Cattle (*Bos taurus*)	Horse (*Equus caballus*)	Goat (*Capra hircus*)	Chamois (*Rupicapra rupicapra*)	Roe deer (*Capreolus capreolus*)	Deer (*Cervus elaphus*)	Chicken (*Gallus gallus domesticus*)	Lizard (*Podarcis muralis*)
TN1	26	humans (< 0.1), wild ungulates (0.09)	4 (15.4)		1 (4.8)				2 (7.7)	19 (73.1)			
TN2	1	humans (3.0), dogs (0.32), horses (0.08), cattle (0.16), gooses (0.16), chickens (0.40)					1 (100.0)						
TN3	71	humans (16.6), wild ungulates (0.06), chickens (0.32), cats (0.32)	2 (2.8)		1 (1.4)			1 (1.4)	4 (5.6)	62 (87.3)		1 (1.4)	
TN4	9	humans (17.0), chickens (1.19), cats (0.24), dogs (0.40), goats (0.40), wild ungulates (0.06)	2 (22.2)					1 (11.1)		5 (55.6)		1 (11.1)	
TN5	1	humans (35.0) , chickens (0.40), dogs (0.56), cats (0.24), wild ungulates (0.06)		1 (100.0)									
TN6	15	wild ungulates (0.09)		1 (6.7)					2 (13.3)	12 (80.0)			
TN7	46	humans (0.2), wild ungulates (0.08)	2 (4.3)						4 (8.6)	37 (80.4)	3 (6.5)		
TN8	1	wild ungulates (0.05), chickens (0.24), ducks (0.32)								1 (100.0)			
TN9	1	humans (< 0.1), dogs (0.40), wild ungulates (0.02)	1 (100.0)										
TN10	3	humans (0.6), dogs (0.32), wild ungulates (0.06)	2 (66.6)							1 (33.3)			
TN11	35	humans (9.3), wild ungulates (0.04), chickens (0.48), dogs (0.48)	1 (2.9)	1 (2.9)						33 (94.3)			
TN12	36	humans (29.4), wild ungulates (0.08), chickens (1.52), dogs (0.24)	2 (5.6)		1 (2.8)					14 (38.9)	19 (52.8)		
TN13	19	humans (2.2), wild ungulates (0.05), dogs (0.48), chickens (0.48)	2 (10.5)							2 (10.5)	15 (78.9)		
TN14	4	humans (1.1), wild ungulates (0.03), dogs (0.08)	1 (2.5)							3 (7.5)			
BL1	2	humans (10.6), chickens (1.59), dogs (0.87)	2 (110.0)										
BL2	3	humans (7.0), cattle (2.39), cats (0.87), chickens (1.99), dogs (0.39)	2 (66.6)			1 (33.3)							
BL3	13	humans (12.5), dogs (0.56), pigeons (1.59), chickens (0.40), horses (0.08)	13 (100.0)										
BL4	1	humans (13.9), dogs (0.24)	1 (100.0)										
BL5	1	humans (13.8), dogs (0.72)	1 (100.0)										
BL6	3	humans (10.7), dogs (0.87), chickens (0.64), cats (0.24)	3 (100.0)										
BL7	4	humans (15.5), dogs (2.94), cattle (2.23), chickens (0.64)	3 (75.0)	1 (25.0)									
BL8	1	humans (15.5), dogs (1.90), cattle (2.23), chickens (0.72)	1 (100.0)										
BL9	3	humans (7.0), dogs (0.79)	1 (33.3)	1 (33.3)									1 (33.3)
Total	299		46 (15.4)	5 (1.7)	3 (1.0)	1 (0.3)	1 (0.3)	2 (0.7)	12 (4.0)	189 (63.2)	37 (12.4)	2 (0.7)	1 (0.3)

*Host density (number of individuals per hectare) at the sampling point was estimated using data from the Global Human Settlement Database^48^ (human population) or from field inspections (other animals), providing a qualitative estimate of host abundance. Other wild animals, such as wild birds, small rodents, or reptiles were not counted.

**Figure 1:**
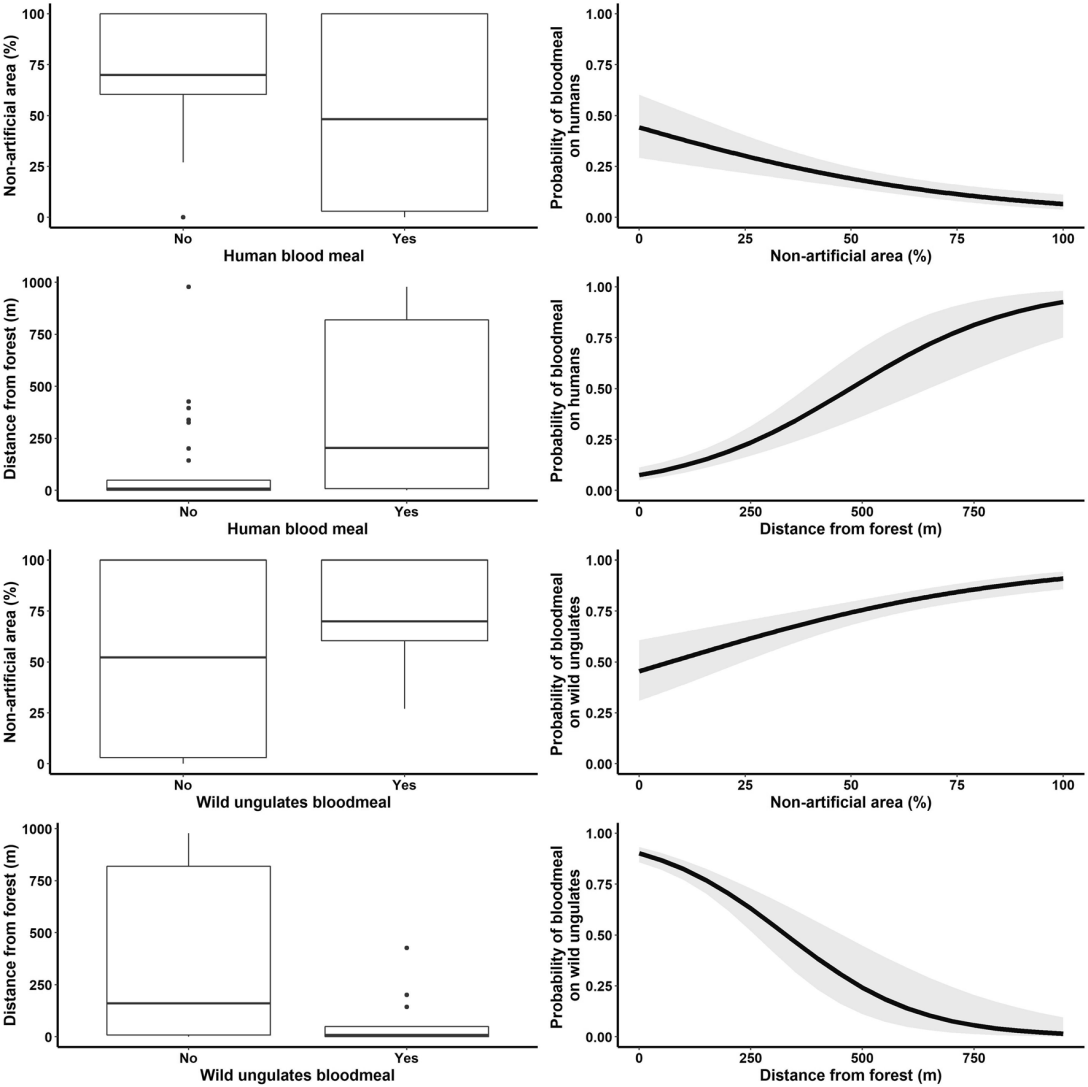
Relationships between significant covariates and the likelihood of a human/ungulate blood meal. Left column: boxplots representing the distribution of the fraction of non-artificial land and the distance of the sampling points from the nearest forest, according to the identified blood meal host. Right column: estimated probability of identifying a blood meal from humans/ungulates (continuous line: average prediction; shaded area: 95% confidence interval). Plots were created using the R libraries “ggplot2” and “gridExtra”.

The results of statistical models (Tables 3 and Fig. 1) showed the probability of identifying blood meals from wild ungulates to be negatively affected (coefficient estimate = − 6.7·10^–3^, SE = 1.2·10^–3^, *p*-value = 4.9∙10^–9^) by distance from a forested area (i.e., wild ungulates were more likely to be identified as blood-meal hosts in mosquitoes collected closer to a forest) and positively affected (coefficient estimate = 2.5·10^–2^, SE = 4.9·10^–3^, *p*-value = 4∙10^–7^) by the fraction of non-artificial land cover (i.e., less urbanized environments). Conversely, blood meals were more likely to be identified as human in less natural areas (coefficient estimate = − 2.4·10^–2^, SE = 5.3·10^–3^, *p*-value = 5.7∙10^–6^) and further from forested sites (coefficient estimate = 5.3·10^–3^, SE = 8.8·10^–4^, *p*-value = 1.4∙10^–9^). Interestingly, both altitude and human population density did not yield a significant relationship for either the human or wild ungulate model (*p*-values > 0.05, see Table 3). Finally, in both cases, the 100 m-buffer model had lower Akaike Information Criterion (AIC) values compared to models with higher distances (250 and 500 m).

**Table 3:** Estimates, standard errors, and *p*-values of univariate coefficients of GLMs assessing the probability of identifying a blood meal from humans and wild ungulates (separate columns).

Explanatory variable	Coefficient Estimate		Standard Error		*p*-value	
	Humans	Wild ungulates	Humans	Wild ungulates	Humans	Wild ungulates
Altitude	2.4·10^–4^	1.6·10^–4^	8.5·10^–4^	7.7·10^–4^	0.78	0.84
Human population	− 5.5·10^–4^	− 4.3·10^–4^	2.8·10^–3^	2.5·10^–3^	0.84	0.86
Fraction of non-artificial land cover (100 m buffer)	− 2.4·10^–2^	2.5·10^–2^	5.3·10^–3^	4.9·10^–3^	5.7·10^–6^	4·10^–7^
Distance from forest	5.3·10^–3^	− 6.7·10^–3^	8.8·10^–4^	1.2·10^–3^	1.4·10^–9^	4.9·10^–9^

## Discussion

In this study, we report the first European data on *Ae. koreicus* feeding patterns in a recently invaded area of northern Italy. Our findings provide evidence of anthropophagy, especially at urban sites in Belluno province, where 96.8% of samples were identified to be of human origin.

To our knowledge, this is one of the first attempts to evaluate the feeding patterns of the invasive mosquito species *Ae. koreicus*, following a preliminary study conducted in 2014^34^. Here, we successfully identified blood meal hosts in 299 out of 352 specimens (84.9%) and detected eleven different host species, suggesting that *Ae. koreicus* might present opportunistic host-feeding behavior, while feeding primarily on mammals. According to our blood meal analyses, it fed mainly on roe deer (63.2%), followed by humans. Roe deer was the primary host in forested sites and humans in urbanized areas. These findings reflect the abundance of the main hosts within the mosquito sampling sites, based on census data. They also suggest that *Ae. koreicus* may feed on the most abundant locally available hosts^35^.

Our statistical analyses highlight that urbanization—measured either as the fraction of artificial land cover or the distance from the nearest forest—is an important factor associated with the likelihood of *Ae. koreicus* feeding on humans or wild ungulates. Interestingly, there was no significant relationship between human density and the likelihood of feeding on either of these hosts. This may be due to the fact that we used available averaged data on the human population density in the surroundings of the sampling point, defined as the square of side 250 m of the Global Human Settlement Database. Future studies aiming at evaluating *Ae. koreicus* feeding behavior should include also the collection of human density data at higher resolution scale. *Aedes koreicus* rarely fed on avian hosts (*N* = 2 blood meals, 0.7%), similarly to previous blood meal analyses of the sibling species *Aedes j. japonicus*^36^. No blood meals were identified from wild birds or pigeons, commonly found in several of the monitored urban sites. However, one study, comparing trap efficacy in China, found that *Ae. koreicus* was attracted and captured by a pigeon-baited trap^37^. Notably, we identified a blood meal from a lizard, underlining the potential for this species to also feed on other taxa. *Aedes albopictus* is the only invasive *Aedes* mosquito reported to feed on ectothermic animals^38^.

No mixed-blood meals were found in our study. This could be the result of this species’ behavior or the molecular method applied^39^. However, mixed-blood meal detection has also been rarely reported in its sibling species *Ae. j. japonicus*^36,40^.

Although some qualitative information was retrieved on the presence of hosts in the capture sites of engorged females, host preference measures, such as the host feeding index or forage ratio, have not been calculated due to the lack of precise quantitative information on all available animal hosts, particularly wild animals and birds^41^. While more details are needed to determine host availability, even a brief survey could provide valuable information. The collected data thus suggest that the feeding pattern is influenced by the abundance or availability of mammalian hosts.

To reduce potential sources of bias that could influence the findings, as site location, collection period, collection method, the samplings were carried out at various time points during the mosquito activity season (May–October), although most were conducted at the peak of mosquito density (July–August)^42^. Moreover, the study included sites with variable habitats and host availability (urban, periurban, rural, or naturally forested). Finally, resting females were caught by different outdoor methods (manual aspiration and traps), and therefore in man-made resting sites located mainly in areas of transition between urban and natural sites.

One limitation of studies on mosquito behavior is the difficulty of collecting large numbers of blood-fed mosquitoes. This problem is particularly common when studying a species in the early stages of invasion, when population density is still limited^43^. The sampling in our study was, however, carried out at sites where *Ae. koreicus* had been established for several years, located in northeastern Italy, which is often reported to have the highest density of the species^6,16,17^. The applied sampling method thus yielded high numbers. One factor affecting potential disease transmission and epidemiology is a mosquito species’ blood-feeding behavior. Opportunistic-feeding mosquitoes may be less likely than specialist-feeding mosquitoes to act as an amplifying vector for a pathogen, given the lower probability of taking blood meals from the same host species. Conversely, they may act as bridge vectors for zoonotic pathogens between a reservoir and a susceptible host species^41^.

The mammalophagic feeding pattern of *Ae. koreicus* suggests that the species may be a potential vector of pathogens that establish transmission cycles among mammals. In urban areas this species feeds almost exclusively on humans, making it a potential vector for human-to-human transmitted arboviruses, as Japanese encephalitis, and Zika, dengue, and chikungunya viruses. In Italy, this species is widespread in hilly and mountainous areas where these diseases (save Japanese encephalitis) have occasionally been diagnosed in travelers (imported human cases), including an autochthonous dengue outbreak sustained by *Ae. Albopictus* occurring in 2020^44^. *Ae. koreicus* may therefore potentially be involved in the transmission of dengue and chikungunya viruses. Since it fed only exceptionally on avian hosts, it may instead have a negligible role as a bridge vector between mammals and birds for pathogens such as West Nile virus.

The potential of a mosquito species to transmit an infectious agent to a new susceptible population is measured by its vectorial capacity. In addition to vector competence and feeding behavior, vectorial capacity is affected by frequency of host contact and vector abundance^45^. Vector abundance is currently not a favorable factor in this setting because the population density of *Ae. koreicus* is still limited in northern Italy^16,17^. This species is, however, able to use a wide variety of habitats and artificial containers and is characterized by a wider period of seasonal activity compared to *Ae. albopictus*^21^. In addition, its observed and predicted spatial spread suggest a high risk of new areas being invaded in a relatively short time, in the absence of control measures^6^. Accordingly, its role and importance as a pest and/or vector could change in the near future.

In conclusion, our results provide the first description of the feeding patterns of *Ae. koreicus* in its invasive range. Further research is needed to determine *Ae. koreicus* vector competence for the various pathogens to which it may be exposed, based on observed feeding patterns.

## Materials and methods

### Study area

**Figure 2:**
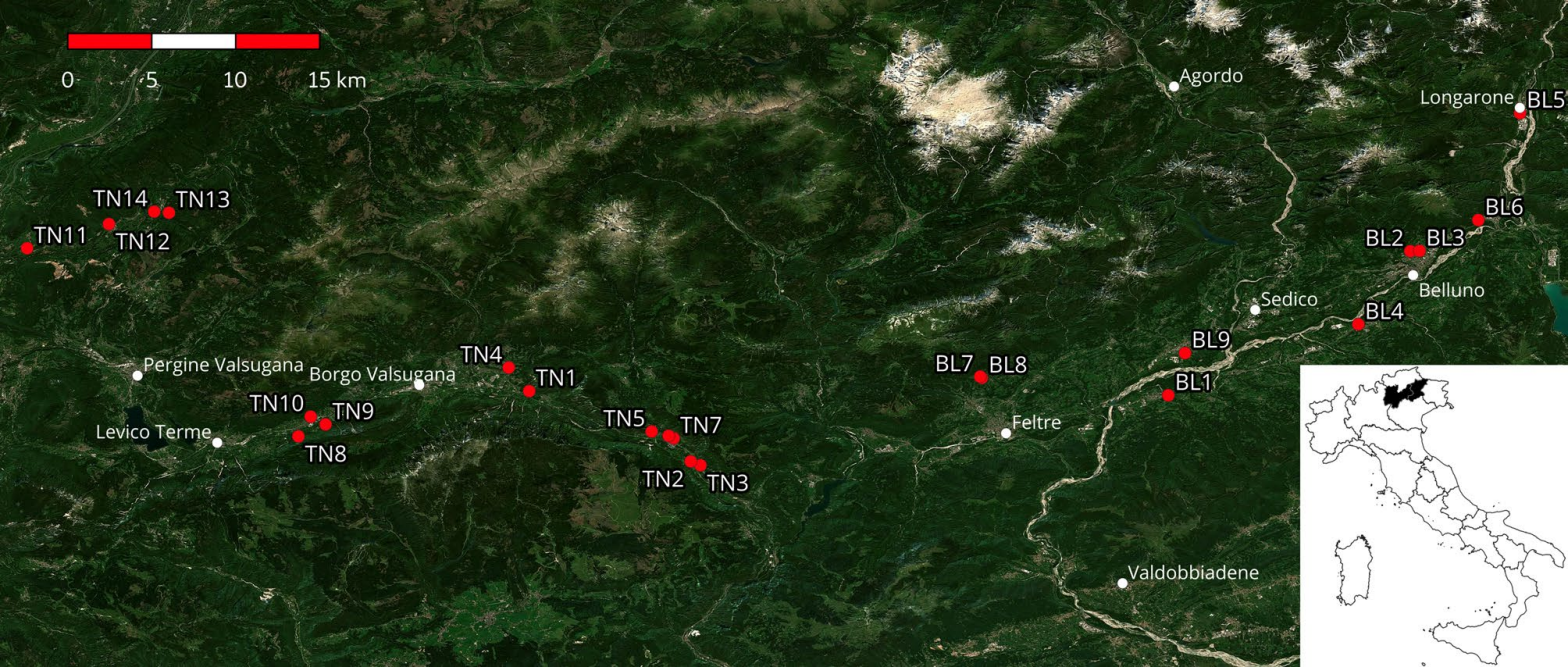
Study area. Points represent the sampling sites marked with the ID number as in Tables 2 and 3. Background satellite image from Sentinel-2 cloudless (https://s2maps.eu), and urban places from OpenStreetMap contributors (https://openstreetmap.org). Map created using QGIS 3.22.

The study area was located in Northeastern Italy (Fig. 2). Specifically, it encompassed 13 municipalities in the Valbelluna (located in Belluno Province), Valsugana, and Cembra valleys (located in Trento Province). The study area has a sub-continental, temperate climate, with cold, often snowy winters and warm, mild summers. Human settlements consist mainly of small villages composed of country houses with private gardens and public parks, all surrounded by forested areas; among the sampled municipalities, only Belluno and Feltre had more than 10,000 inhabitants.

### Host survey

The presence and abundance of domestic animal hosts in each site were estimated through a door-to-door census. As the flight range of *Ae. koreicus* is unknown, a field inspection was performed within a 200-m radius of the sampling site, corresponding to the average flight distance of *Ae. albopictus* recorded in a study conducted in Italy^46^. The survey was carried out once in 2020. Residents were asked if they owned animals (dogs, cats, farm animals) and how many they had or, where possible, they were counted directly by the study team (visual inspection). The presence of wild ungulates was estimated according to data provided by the Forestry and Fauna Service—Wildlife Office of the Autonomous Province of Trento. The wild ungulate census was carried out in spring by visual inspection along transects, and repeated three times by hunters and personnel of the wildlife management provincial office.^47^. The average number of roe deer, red deer, and chamois in 2020 was considered for the analyses. Collected information was used to qualitatively estimate potential host availability in the sampling areas. Human population density in the areas surrounding the sampling point was estimated using the Global Human Settlement Database (GHS Data)^48^.

### Collection of Aedes koreicus and blood meal analysis

**Figure 3:**
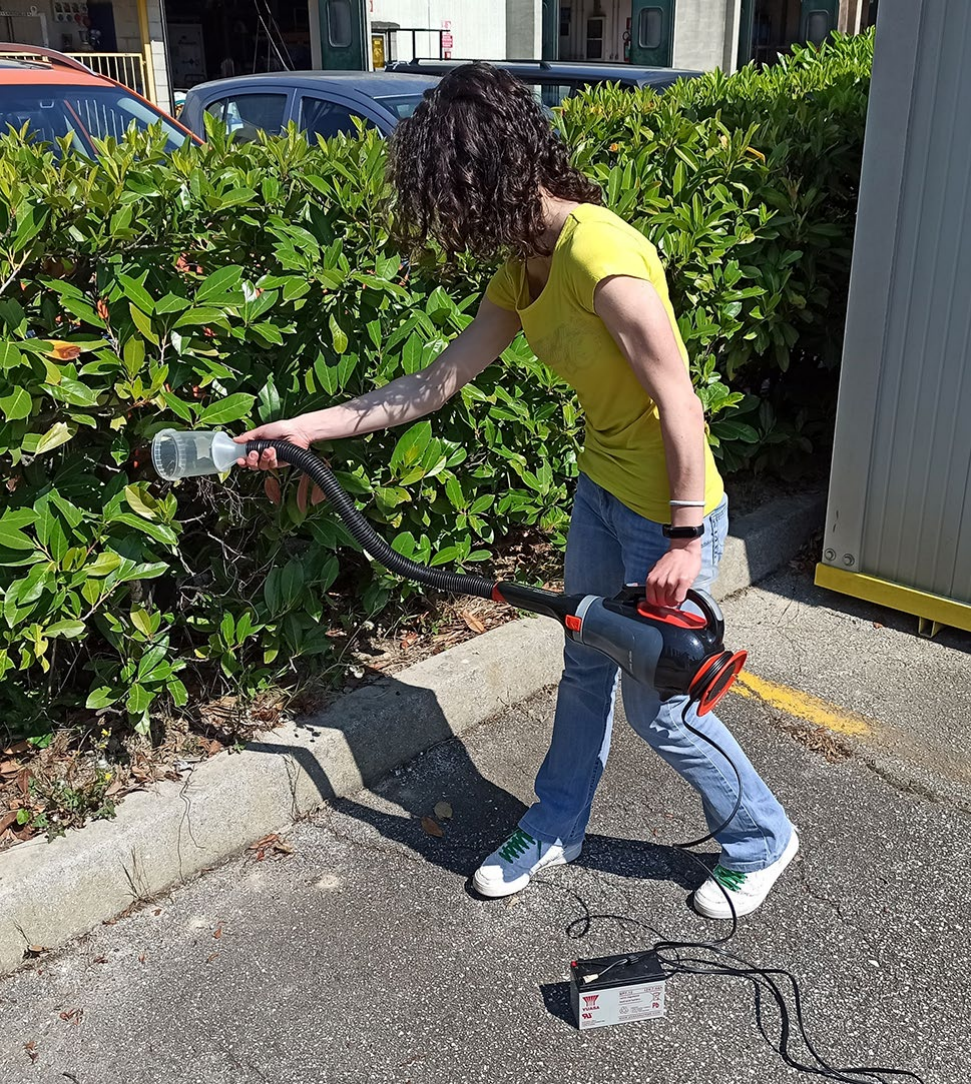
Home-built handheld aspirator (a modified handheld vacuum).

Sampling was carried out from 2013 to 2020 (from May to October) with different frequencies in the various years; most collections were made in 2020 (20 collections) and just one in 2019. In total, 23 different sites were sampled where *Ae. koreicus* were known to be present: 14 in Trento and 9 in Belluno Province, respectively (Table 1 and Supplementary Table S1 online), with altitudes ranging from 234 to 775 m a.s.l.^6,16^. Engorged mosquitoes were collected in public and private houses, garden centers, cemeteries, and from periurban dry-stone walls using a home-built handheld aspirator (a modified handheld vacuum) (Fig. 3). Mosquitoes were aspirated from shady areas under vegetation, walls, and catch basins. In addition, all engorged females collected during routine invasive mosquito surveillance were used for the analyses. In this surveillance, BG-sentinel traps (Biogents AG, Regensburg, Germany) baited with a BG-Lure cartridge (Biogents) were activated for 24 h fortnightly. Immediately after collection, each sample was placed in a cooler, transported to the laboratory, and stored at − 80 °C until molecular analysis.

Sampled mosquitoes were identified at species level according to Montarsi et al.^21^ and ECDC guidelines for invasive mosquito surveillance in Europe^49^. Blood-fed females were isolated from collected mosquitoes to identify the blood meal host.

DNA of single blood-fed mosquito samples, collected from 2013 to 2016, was extracted using Microlab Starlet automated liquid-handling workstations (Hamilton), using a MagMAX Pathogen RNA/DNA kit (Applied Biosystems, USA), according to the manufacturer’s instructions. DNA of a single abdomen of blood-fed mosquitoes, collected from 2017 to 2020, was extracted using QIAamp DNA Investigator kit tissues (Qiagen, Germany), following the manufacturer’s protocol. All samples were analyzed using a nested PCR with a specific set of primers targeting the vertebrate mitochondrial cytochrome c oxidase subunit I (COI) gene, as previously described^50^. The first PCR reaction was carried out in a total volume of 50 μl, containing 2 units of AmpliTaq Gold DNA Polymerase (Applied Biosystem, USA), 5 μl of 10X Buffer, 2.5 mM of MgCl2, 0.2 mM of each dNTP, 2.5 μl of DMSO, 0.2 mM of primers M13BCV-FW (5’-TGT AAA ACG ACG GCC AGT HAA YCA YAA RGA YAT YGG-3’) and BCV-RV1 (5’-GCY CAN ACY ATN CCY ATR TA-3’), and 5 μl of extracted DNA. The second PCR reaction was carried out in a total volume of 50 μl containing 2 units of AmpliTaq Gold DNA Polymerase (Applied Biosystem, USA), 5 μl of 10X Buffer, 2.0 mM of MgCl2, 0.2 mM of each dNTP, 2.5 μl of DMSO, 0.4.mM of primers M13 (5’-GTA AAA CGA CGG CCA GTG-3’) and BCV-RV2 (5’-ACY ATN CCY ATR TAN CCR AAN GG-3’), and 1 μl of the PCR products obtained during the first amplification step. The thermal profile of the first PCR consisted of activation at 95 °C for 10 min, followed by 40 cycles at 94 °C for 40 s, 45 °C for 40 s, and 72 °C for 1 min, with a final extension step of 7 min at 72 °C. The thermal profile of the second PCR consisted of activation for 10 min at 95 °C followed by 16 cycles of a touchdown protocol at 94 °C for 40 s, decreasing the annealing temperature from 60 °C to 45 °C for 40 s (1 °C/cycle), followed by 72 °C for 1 min. Then, 30 cycles at 94 °C for 40 s, 45 °C for 40 s, and 72 °C for 1 min, with a final extension step of 7 min at 72 °C. Negative controls were included during the extraction and amplification stages to confirm avoidance of contamination.

The amplicons were sequenced in both directions using a 16-capillary ABI PRISM 3130xl Genetic Analyzer (Applied Biosystems, USA). To identify the blood meal host species, nucleotide sequences were compared with representative sequences available in the GenBank database using the Basic Local Alignment Search Tool (BLAST). Positive identification was made when > 97% identity was attained between the query and subject sequence.

### Statistical analysis

As most of the identified hosts were either humans or wild ungulates (see Results), we investigated how the probability of feeding on these two host groups was affected by different abiotic factors. Specifically, we considered two binary response variables indicating whether or not the blood meal was acquired from a human/wild ungulate host. We developed univariate (i.e., with only one explanatory variable) generalized linear models (GLMs) with a binomial-distributed error structure, considering in turn, for each response variable, the following four explanatory covariates: (i) the altitude of the sampling point; (ii) the human population density in the area surrounding the sampling point, defined as 250 m square units, as per the Global Human Settlement Database^48^; (iii) the percentage of non-artificial land cover within different buffers (100, 250 and 500 m radius from the sampling point), as per the Corine Land Cover dataset (defined as the sum of the fractions of agricultural and forested areas)^51^; the distance associated with the model with the lowest AIC value was then selected; (iv) the minimum distance of the sampling point from the nearest pixel labeled as forest, according to the Corine category. All analyses, including plot creation, was performed using R v4.0.2^52^ and “tidyverse”, “ggplot2”, and “gridExtra” libraries.

Map in Fig. 1 was generated by QGIS 3.22 using Sentinel-2 cloudless as background satellite image and urban places from OpenStreetMap database^53–55^.

## Supplementary Information

Supplementary Information.

## Data Availability

All data generated or analysed during this study are included in this published article.

## References

[1] Tanaka K, Mizusawa K, Saugstad ES (1979). A revision of the adult and larval mosquitoes of Japan (including the Ryukyu Archipelago and the Ogasawara islands) and Korea (Diptera: Culicidae). Contrib. Am. Entomol. Inst..

[2] Bezzhonova OV, Patraman IV, Ganushkina LA, Vyshemirskiĭ OI, Sergiev VP (2014). The first finding of invasive species *Aedes (Finlaya) koreicus* (Edwards, 1917) in European Russia. Med. Parazitol. (Mosk)..

[3] Versteirt V, Pecor JE, Fonseca DM, Coosemans M, Van Bortel W (2012). Confirmation of *Aedes koreicus* (Diptera: Culicidae) in Belgium and description of morphological differences between Korean and Belgian specimens validated by molecular identification. Zootaxa.

[4] Kaufman MG, Fonseca DM (2014). Invasion biology of *Aedes japonicus japonicus* (Diptera: Culicidae). Annu. Rev. Entomol..

[5] Marcantonio M (2016). First assessment of potential distribution and dispersal capacity of the emerging invasive mosquito *Aedes koreicus* [*Hulecoeteomyia koreica*] in Northeast Italy. Parasit. Vectors..

[6] Marini G (2019). First report of the influence of temperature on the bionomics and population dynamics of *Aedes koreicus*, a new invasive alien species in Europe. Parasit. Vectors..

[7] European Centre for Disease Prevention and Control and European Food Safety Authority. Mosquito maps [internet]. Stockholm: ECDC; 2021. https://ecdc.europa.eu/en/disease-vectors/surveillance-and-disease-data/mosquito-maps.

[8] Capelli G (2011). First report in Italy of the exotic mosquito species *Aedes* (*Finlaya*) *koreicus*, a potential vector of arboviruses and filariae. Parasit. Vectors..

[9] Versteirt V (2012). Bionomics of the established exotic mosquito species *Aedes koreicus* in Belgium Europe. J. Med. Entomol..

[10] Pfitzner WP, Lehner A, Hoffmann D, Czajka C, Becker N (2018). First record and morphological characterization of an established population of *Aedes* (*Hulecoeteomyia*) *koreicus* (Diptera: Culicidae) in Germany. Parasit. Vectors..

[11] Kurucz K (2016). Emergence of *Aedes koreicus* (Diptera: Culicidae) in an urban area, Hungary, 2016. Parasitol. Res..

[12] Suter T (2015). First report of the invasive mosquito species *Aedes koreicus* in the Swiss-Italian border region. Parasit. Vectors..

[13] Kalan K, Šušnjar J, Ivović V, Buzan E (2017). First record of *Aedes koreicus* (Diptera, Culicidae) in Slovenia. Parasitol. Res...

[14] Fuehrer HP (2020). Monitoring of alien mosquitoes in Western Austria (Tyrol, Austria, 2018). PLoS Negl. Trop. Dis..

[15] Andreeva YV (2021). First record of the invasive mosquito species *Aedes koreicus* (Diptera, Culicidae) in the Republic of Kazakhstan. Parasite.

[16] Montarsi F (2015). Current distribution of the invasive mosquito species, *Aedes koreicus* [*Hulecoeteomyia koreica*] in northern Italy. Parasit. Vectors..

[17] Gradoni F (2021). Geographical data on the occurrence and spreading of invasive *Aedes* mosquito species in Northeast Italy. Data Brief..

[18] Negri A (2021). Evidence for the spread of the alien species *Aedes koreicus* in the Lombardy region Italy. Parasit Vectors..

[19] Miyagi I (1971). Notes on the *Aedes* (*Finlaya*) *chrysolineatus* subgroup in Japan and Korea (Diptera: Culicidae). Trop. Med..

[20] Medlock JM (2015). An entomological review of invasive mosquitoes in Europe. Bull. Entomol. Res..

[21] Montarsi F (2013). Distribution and habitat characterization of the recently introduced invasive mosquito *Aedes koreicus* [*Hulecoeteomyia koreica*], a new potential vector and pest in north-eastern Italy. Parasit. Vectors..

[22] Tatem AJ, Hay SI, Rogers DJ (2006). Global traffic and disease vector dispersal. Proc. Natl. Acad. Sci. USA.

[23] La Casse, W.J. & Yamaguti, S. *Mosquito fauna of Japan and Korea.* (In Part I and II. Edited by Corps of Engineers. U.S. ARMY, 1950).

[24] Baldacchino F (2017). Weak larval competition between two invasive mosquitoes *Aedes koreicus* and *Aedes albopictus* (Diptera: Culicidae). J. Med. Entomol..

[25] Shestakov VI, Mikheeva AI (1966). Contribution to study of Japanese encephalitis vectors in Primorye region. Med. Parazitol..

[26] Gutsevich, A.V. & Monchadskii Stackel’berg, A.A. Mosquitoes, family culicidae. in (ed Bykhovskii, B. E.) *Fauna of the U.S.S.R.-Diptera*. Vol. III, No. 4. Leningrad: Acad. Sci. SSSR Zool. Inst. 73–405 (1971).

[27] Miles JA (1964). Some ecological aspects of the problem of arthropod-borne animal viruses in the Western Pacific and South-East Asia Regions. Bull. World Health Organ..

[28] Feng LC (1930). Experiments with *Dirofilaria immitis* and local species of mosquitoes in Peiping North China. Ann. Trop. Med. Parasit..

[29] Montarsi F (2015). Development of *Dirofilaria immitis* within the mosquito *Aedes* (*Finlaya*) *koreicus*, a new invasive species for Europe. Parasit. Vectors..

[30] Ciocchetta S (2018). The new European invader *Aedes (Finlaya) koreicus*: a potential vector of chikungunya virus. Pathog. Glob. Health..

[31] Jansen S (2021). Vector competence of the invasive mosquito species *Aedes koreicus* for arboviruses and interference with a novel insect specific virus. Viruses.

[32] Fikrig K (2021). The effects of host availability and fitness on *Aedes albopictus* blood feeding patterns in New York. Am. J. Trop. Med. Hyg..

[33] Hsiao, T., Bohart R.M. The mosquitoes of Japan and their medical importance: U.S. NAVMED 1095. Bureau of Medicine and Surgery Navy Department, Washington DC, (1946).

[34] Montarsi, F., *et al.* Current knowledge on the distribution and biology of the recently introduced invasive mosquito *Aedes koreicus* (Diptera: Culicidae). *Atti Accad. Naz. Ital. Entomol.* Anno LXII, 169–1742, (2014).

[35] Takken W, Verhulst NO (2013). Host preferences of blood-feeding mosquitoes. Annu. Rev. Entomol..

[36] Schönenberger AC (2016). Host preferences in host-seeking and blood-fed mosquitoes in Switzerland: Host preferences in mosquitoes. Med. Vet. Entomol..

[37] Wang ZM (2012). Biting activity and host attractancy of mosquitoes (Diptera: Culicidae) in Manzhouli China. J. Med. Entomol..

[38] Delatte H (2010). Blood-feeding behavior of *Aedes albopictus*, a vector of Chikungunya on La Réunion. Vector-Borne Zoonot..

[39] Kent RJ (2009). Molecular methods for arthropod bloodmeal identification and applications to ecological and vector-borne disease studies. Mol. Ecol. Resour..

[40] Cebrián-Camisón, S., Martínez-de la Puente, J. & Figuerola, J. A Literature review of host feeding patterns of invasive *Aedes* mosquitoes in Europe. *Insects*, **11(12),** 848, 10.3390/insects11120848 (2020). 10.3390/insects11120848 PMC7760726 33260438

[41] Fikrig K, Harrington LC (2021). Understanding and interpreting mosquito blood feeding studies: the case of *Aedes albopictus*. Trends Parasitol..

[42] Baldacchino F (2017). A 2-yr Mosquito Survey Focusing on *Aedes koreicus* (Diptera: Culicidae) in Northern Italy and Implications for Adult Trapping. J. Med. Entomol..

[43] Liebhold AM, Tobin PC (2008). Population ecology of insect invasions and their management. Annu. Rev. Entomol..

[44] Barzon L (2021). Autochthonous dengue outbreak in Italy 2020: clinical, virological and entomological findings. J. Travel Med..

[45] Kramer LD, Ciota AT (2015). Dissecting vectorial capacity for mosquito-borne viruses. Curr. Opin. Virol..

[46] Marini F, Caputo B, Pombi M, Tarsitani G, della Torre A (2010). Study of *Aedes albopictus* dispersal in Rome, Italy, using sticky traps in mark-release-recapture experiments. Med. Vet. Entomol..

[47] Analisi delle consistenze e dei prelievi di ungulati, tetraonidi e coturnice, Stagione venatoria 2020. https://forestefauna.provincia.tn.it/content/download/12964/232268/file/relazione%202019%20Web.pdf.

[48] Schiavina, M., Freire, S. & MacManus, K. GHS-POP R2019A - GHS population grid multitemporal (1975-1990-2000-2015). European Commission, Joint Research Centre (JRC) . 10.2905/0C6B9751-A71F-4062-830B-43C9F432370F PID: http://data.europa.eu/89h/0c6b9751-a71f-4062-830b-43c9f432370f (2019).

[49] European Centre for Disease Prevention and Control. Guidelines for the surveillance of invasive mosquitoes in Europe. Stockholm: ECDC; 2012. https://www.ecdc.europa.eu/en/publications-data/guidelines-surveillance-invasive-mosquitoes-europe. 22971331

[50] Alcaide, M., *et al.* Disentangling vector-borne transmission networks: a universal DNA barcoding method to identify vertebrate hosts from arthropod bloodmeals. *PloS one.* **4(9)**, e7092, 10.1371/journal.pone.0007092 (2009). Corrections: 10.1371/annotation/1e67b1e0-a356-4b3a-8c4c-03037ccfe1f710.1371/annotation/ce5bff53-4638-4931-aa68-904b74db4b20 10.1371/journal.pone.0007092 PMC2740869 19768113

[51] Copernicus Land Monitoring Service. https://land.copernicus.eu/pan-european/corine-land-cover.

[52] R Core Team, 2020. R: A language and environment for statistical computing. https://www.R-project.org/.

[53] QGIS (Quantum GIS) 3.22 version. https://github.com/qgis/QGIS/releases/tag/final-3_22_1 in https://qgis.org. Accessed 22 April 2022.

[54] Sentinel-2. https://s2maps.eu. Accessed 22 April 2022.

[55] OpenStreetMap. https://openstreetmap.org. Accessed 22 April 2022.

