# Supplementary material for: First report of the blood-feeding pattern in Aedes koreicus, a new invasive species in Europe
Source: Sci Rep. 2022 Sep 21;12:15751. doi: 10.1038/s41598-022-19734-z (PMC9492761; doi:10.1038/s41598-022-19734-z)
Supplement: Supplementary file 1 — Supplementary Information. [file 41598_2022_19734_MOESM1_ESM.pdf]

First report of the blood feeding pattern in *Aedes koreicus*, a new invasive species in Europe

Fabrizio Montarsi[1*], Fausta Rosso[2], Daniele Arnoldi[2], Silvia Ravagnan[1], Giovanni Marini[2], Luca Delucchi[2], Roberto Rosà[2,3], & Annapaola Rizzoli[2].

[1]Istituto Zooprofilattico Sperimentale delle Venezie, Legnaro, Padua, Italy

[2]Research and Innovation Centre, Fondazione Edmund Mach, San Michele all'Adige (TN), Italy.

[3]Center Agriculture Food Environment, University of Trento, San Michele all’Adige (TN), Italy

Supplementary Table S1. List of sampling sites with geographical details (DD=decimal degree), environmental description, collection methods (BG-S=BG-sentinel trap baited with BG-Lure), and engorged females collected (2013-2020).

ID site	Municipality	Province	Latitude (DD)	Longitude (DD)	type of site	landscape Corine Land Cover	N. of collections	collection methods	fed females
TN1	Castel Ivano	Trento	46.048667	11.540833	forest	Agricultural: 18.85% Forest: 65.26%	11	aspiration/BG-S	34
TN2	Grigno	Trento	45.994792	11.664571	periurban	Artificial: 42.73% Agricultural: 49.56%	1	BG-S	1
TN3	Grigno	Trento	45.991875	11.672044	forest/ periurban	Artificial: 42.96% Forest: 51.69%	34	aspiration/BG-S	83
TN4	Castel Ivano	Trento	46.066722	11.525146	periurban	Artificial: 62.60% Forest: 16.25%	13	aspiration	16
TN5	Grigno	Trento	46.017710	11.634680	forest/ periurban	Artificial: 30.68% Forest: 49.69%	1	aspiration	1
TN6	Grigno	Trento	46.014430	11.647692	forest	Artificial: 8.96% Agricultural: 33.54% Forest: 35.82%	6	aspiration	16
TN7	Grigno	Trento	46.012566	11.651511	forest	Artificial: 9.51% Agricultural: 26.4% Forest: 18.2%	11	aspiration	46
TN8	Novaledo	Trento	46.014006	11.363997	forest/ agricultural	Agricultural: 48.73% Forest: 51.28%	1	aspiration	1
TN9	Novaledo	Trento	46.023157	11.384775	forest/ agricultural	Agricultural: 64.08% Forest: 22.2%	1	aspiration	1
TN10	Novaledo	Trento	46.029123	11.373348	forest/ agricultural	Artificial: 32.63% agricultural: 9.07% Forest: 43.57%	2	aspiration	3
TN11	Giovo	Trento	46.158150	11.156060	forest/ periurban	Artificial: 63.46% Agricultural: 36,54%	5	aspiration	37
TN12	Cembra Lisignago	Trento	46.176720	11.218780	forest/ periurban	Artificial: 32.45% Forest: 45.87%	3	aspiration	37
TN13	Segonzano	Trento	46.185350	11.264790	forest/ periurban	Artificial: 4.41% Agricultural: 68.33% Forest: 8.90%	5	aspiration	26
TN14	Segonzano	Trento	46.186240	11.253440	forest/ agricultural	Artificial: 0.89% Agricultural: 77.98%	2	aspiration	4
BL1	Borgo Valbelluna	Belluno	46.045500	12.030598	farm	Artificial: 24.03% Agricultural: 52.33% Forest: 8.42%	1	aspiration	2

BL2	Belluno	Belluno	46.155972	12.216055	garden center	Artificial: 57.66% Agricultural: 42.34%	1	aspiration	5
BL3	Belluno	Belluno	46.156167	12.223083	cemetery	Artificial: 45.38% Agricultural: 54.63%	5	aspiration	23
BL4	Limana	Belluno	46.099889	12.176222	cemetery	Artificial: 29.65% Agricultural: 46.54% Forest: 2.14%	1	aspiration	2
BL5	Longarone	Belluno	46.261709	12.300139	cemetery	Artificial: 30.91% Agricultural: 69.10%	1	aspiration	1
BL6	Ponte nelle Alpi	Belluno	46.179773	12.268085	sporting center	Artificial: 24.03% Agricultural: 52.33% Forest: 8.42%	1	aspiration	5
BL7	Feltre	Belluno	46.059814	11.886404	kennel	Artificial: 46.96% Agricultural: 22.51% Forest: 26.32%	3	BG-S	4
BL8	Feltre	Belluno	46.058852	11.887635	periurban	Agricultural 100%	1	BG-S	1
BL9	Santa Giustina	Belluno	46.077799	12.043377	train station	Artificial: 64.09% Agricultural: 14.26%	2	BG-S	3
